# Effect of adjuvant treatment with Xiyanping injection on the prognosis of viral encephalitis in children: a multicenter retrospective study

**DOI:** 10.3389/fphar.2025.1632728

**Published:** 2025-10-30

**Authors:** Wen Tian, Yang Chen, Huazhang Liu, Danning Wen, Zhe Wang, Ying Li, Li Liu, Xiangna Yang, Xueyan Ma, Yuanyuan Zhang, Chengjie Ma, Rongbing Wang, Qiaozhi Yang, Yibing Yan, Yukun Zhang, Xiaohong Gu, Wei Zhang

**Affiliations:** ^1^ College of Traditional Chinese Medicine, Beijing University of Chinese Medicine, Beijing, China; ^2^ Department of Pediatrics, Liaocheng People’s Hospital, Liaocheng, Shandong, China; ^3^ Department of Pediatrics, The First Affiliated Hospital of Shandong First Medical University, Jinan, Shandong, China; ^4^ Shandong Engineering and Technology Research Center for Pediatric Drug Development, Shandong, China; ^5^ Wuhan Jinyintan Hospital Infected Ward One, Wuhan, Hubei, China; ^6^ Department of Infectious Diseases, Tianjin Second People’s Hospital, Tianjin, China; ^7^ Department of Neurology, Shanghai Children’s Medical Center, School of Medicine, Shanghai JiaoTong University, Shanghai, China; ^8^ Department of Pediatrics, Guangzhou Women and Children’s Medical Center, Guangzhou, China; ^9^ National Key Laboratory of Intelligent Tracking and Forecasting for Infectious Diseases, Beijing Ditan Hospital, Capital Medical University, Beijing, China; ^10^ Center for Infectious Diseases, Beijing Ditan Hospital, Capital Medical University, Beijing, China; ^11^ National Center for Infectious Diseases(Beijing), Beijing Ditan Hospital, Capital Medical University, Beijing, China

**Keywords:** complementary alternative medicine, viral encephalitis (VE), Xiyanping injection, prognosis, economic burden

## Abstract

**Background:**

Viral encephalitis (VE), a central nervous system disorder with high mortality and disability rates, poses a serious threat to childhood development. Xiyanping injection (XYPI), an andrographolide sulfonate preparation widely used in China, exhibits anti-inflammatory, antiviral, antitumor, antibacterial and neuroprotective properties.

**Methods:**

We conducted a retrospective study of 635 pediatric inpatients with VE who were hospitalized at seven medical centers in China between January 2015 and December 2021. Risk factors for poor prognosis were compared between inpatients treated with XYPI (n = 480) and those without XYPI treatment (n = 155). Propensity score matching was performed to reduce potential confounding. Clinical symptoms, hospitalization costs, complications and sequelae were evaluated simultaneously.

**Results:**

Multivariate Logistic regression identified XYPI treatment as an independent protective factor for poor prognosis (odds ratio [OR] = 0.251, 95% confidence interval [CI]: 0.113–0.559, p < 0.001). XYPI significantly shortened the duration of fever and headache, reduced hospitalization costs, and lower the incidences of respiratory infections, myocardial injury, and sequelae (all p < 0.05).

**Conclusion:**

Adjuvant XYPI therapy may improve clinical outcomes and reduce the economic burden in pediatric VE; however, randomized trials are warranted to validate these findings.

## 1 Introduction

Viral encephalitis (VE), a widespread infectious disease of the central nervous system, accounts for approximately 20%–50% of encephalitis cases worldwide (Vora et al., 2014). Characterized by high mortality and disability rates, VE represents a major global health burden. It is defined as inflammation of the brain parenchyma caused by neurotropic viruses and presents with diverse clinical manifestations, ranging from mild symptoms to diffuse or focal neurological deficits. Patients may also exhibit nonspecific viral prodromes, including fever, headache, nausea, and vomiting (Venkatesan et al., 2013). Importantly, VE is frequently associated with severe and long-term sequelae such as irreversible neurological damage and cognitive impairment (Mcgrath et al., 1997; Mailles and Stahl, 2009).

The diagnosis of VE requires a comprehensive approach that integrates clinical features, laboratory testing, and epidemiological history. The gold standard for confirmation is the detection of specific viral antigens, antibodies, or nucleic acids in cerebrospinal fluid or brain tissue (Feng et al., 2020). Despite advances in diagnostic techniques, however, the causative virus remains unidentified in 30%–62% of clinically confirmed cases (Yong et al., 2023). A wide range of viruses—including enteroviruses, herpes simplex virus (HSV), epstein–barr virus, human immunodeficiency virus, and other unidentified agents—have been implicated in VE (Vora et al., 2014; George et al., 2014). Among children, HSV encephalitis and enterovirus encephalitis are the most common etiologies (Bloch et al., 2023; Parvez and Ohtsuki, 2022). Pediatric VE imposes a considerable economic burden on both health systems and families (Li et al., 2023).

Supportive care remains the cornerstone of VE management, and approximately 20% of patients require intensive care unit admission (Thakur et al., 2013). Complications such as deep vein thrombosis and epilepsy often demand concurrent treatment (Bradshaw and Venkatesan, 2019; Steiner et al., 2005). Despite the availability of antiviral therapy, patients with HSV encephalitis continue to experience mortality rates of up to 28% and frequently develop severe neurological sequelae (Bradshaw and Venkatesan, 2016). Overall, VE exhibits a mortality rate ranging from 1% to 90% owing to the lack of specific antiviral agents (Ludlow et al., 2016).

In the theoretical framework of traditional Chinese medicine (TCM), VE is not recognized as an independent disease entity. Based on its key clinical manifestations and neurological dysfunction, however, it is classified within the broader category of *Warm Disease.* The TCM pathogenesis of VE is considered multifactorial, involving disturbances of multiple organ systems, particularly the brain, heart, lungs, liver, and kidneys. This holistic perspective aligns with modern biomedical evidence showing that inadequate viral clearance at the primary infection site can allow dissemination to the central nervous system and other organs, leading to multisystem complications (Yang et al., 2023).

The TCM etiology of VE can be interpreted from two principal perspectives. First, it is attributed to exposure to “epidemic toxins,” a concept closely paralleling the modern biomedical notion of pathogens. Second, it arises from a deficiency of *healthy Qi,* reflecting an intrinsic weakness in the body’s ability to resist pathogenic invasion. This deficiency, together with an imbalance between Yin and Yang, creates systemic dysfunction that allows pathogenic toxins to ascend and invade the brain orifices, thereby inducing encephalitis (Jia et al., 2016). Emerging evidence supports these concepts: the severity of HSV encephalitis in children is strongly associated with monogenic inborn errors in the Toll-like receptor 3–dependent signaling pathway, which impair the production of key antiviral cytokines such as Interferon-beta and Interferon-lambda—a mechanistic defect analogous to the TCM concept of *healthy Qi* deficiency (Zhang, 2020). Moreover, neurotropic viruses can disrupt the blood–brain barrier and trigger both adaptive immune responses and trained immunity, culminating in cytokine storm and aggravated neuroinflammation, a process that mirrors the TCM doctrine of Yin–Yang imbalance (Matthews et al., 2025).

TCM provides multiple therapeutic strategies for VE, including herbal decoctions, proprietary Chinese patent medicines, and acupuncture (Wang, 2006). Clinical evidence indicates that formulas such as Changpu Yujin Decoction and Baihu Decoction significantly enhance treatment efficacy in VE (Rao and Han, 2023; Wang et al., 2016). A systematic review of 23 clinical trials demonstrated that Xingnaojing injection combined with conventional therapy improved cure rates, shortened symptom recovery time, and reduced mortality (Cao et al., 2019). Similarly, a meta-analysis of 1,045 cases reported that Angong Niuhuang Pill, when used as an adjunctive therapy, increased the overall effectiveness rate by 17% (Huang et al., 2024). Our previous work also showed that proprietary Chinese medicines effectively improved clinical symptoms and reduced sequelae in pediatric VE (Wang et al., 2024). Acupuncture, used as an adjuvant therapy, has likewise demonstrated clinical benefits; in a rat model of herpes simplex encephalitis, acupuncture improved spatial learning and memory impairments (Jin et al., 2024). Furthermore, the antiviral activities of specific herbal extracts are being increasingly validated. For example, Rhodiola extract disrupts the binding and stability of Japanese encephalitis virus, whereas Artemisia argyi extract damages the integrity of HSV particle membranes, offering new potential therapeutic strategies for viral infections (Lai et al., 2024; Liu et al., 2023).

Xiyanping injection (XYPI), a well-established injectable preparation with a long history of clinical use in China, exhibits broad therapeutic activities, including potent anti-inflammatory, antiviral, and antibacterial effects. XYPI is primarily composed of andrographolide sulfonate (AS). Using high-performance liquid chromatography, Zhan et al. identified four key metabolites of XYPI: andrographolide sulfate ester A, andrographolide sulfate ester B, andrographolide sulfate ester C, and 9-dehydro-17-hydroxyandrographolide (Zhan et al., 2012). Accumulating evidence also indicates that XYPI has additional pharmacological effects, including antitumor and neuroprotective activities. (Lu et al., 2019).

XYPI has been incorporated into the Chinese Pediatric Association guidelines as a first-line therapy for several pediatric infectious diseases, such as viral pneumonia, acute undifferentiated febrile illness, hand-foot-and-mouth disease, and influenza-like illnesses (Wang et al., 2019; Yang et al., 2024). Pharmacokinetic studies in rat models have revealed extensive tissue distribution of XYPI metabolites, with quantifiable concentrations detected in the brain, heart, kidneys, liver, lungs, and spleen (Bera et al., 2014). Moreover, a large-scale real-world clinical study conducted in China demonstrated that XYPI is an effective therapeutic agent for VE, with enhanced efficacy observed when used in combination with conventional chemical drugs (Sheng et al., 2022).

Despite significant advances in neuropharmacological research on XYPI, high-quality clinical evidence supporting its use as an adjuvant therapy for pediatric VE remains scarce. This retrospective study therefore aims to comprehensively assess the benefit–risk profile of XYPI-augmented regimens in the management of pediatric VE, thereby providing an evidence-based foundation for optimizing neuroprotective strategies in this vulnerable population.

## 2 Materials and methods

### 2.1 Drug information

XYPI contains only AS and water for injection, with no additional excipients. It has been approved by the China National Medical Products Administration under drug approval number WS-10863-(ZD-0863)-2002 and national drug registration number Z20026249. AS is primarily extracted from *Andrographis paniculata (Burm. f.) Nees* (Acanthaceae; Andrographidis herba). In this study, all XYPI reagents were supplied by Jiangxi Qingfeng Pharmaceutical Co.,Ltd. The contents of total sulfonates and 17-hydroxy-9-dehydroandrographolide-19-sulfate sodium were quantitatively determined for quality control. Furthermore, chromatographic fingerprint analysis confirmed that their retention times were identical to those of the corresponding reference peaks. The product inspection reports and batch-specific documentation for XYPI are provided in the Supplementary Material.

### 2.2 Population

Between 2015 and 2021, we conducted a retrospective analysis of 919 pediatric inpatients diagnosed with VE across multiple hospitals, including Beijing Ditan Hospital, Liaocheng Children’s Hospital, the First Affiliated Hospital of Shandong First Medical University, Guangzhou Women and Children’s Medical Center, Shanghai Children’s Medical Center, Wuhan Jinyintan Hospital, and Tianjin Second People’s Hospital.

The inclusion criteria were: (1) age between 0 and 18 years old (2) a diagnosis of VE consistent with the consensus statement of the International Encephalitis Consortium (Venkatesan et al., 2013).

The exclusion criteria were: (1) coexisting bacterial or autoimmune encephalitis; (2) concomitant use of other TCM adjuvant therapies; (3) death on the day of hospitalization; (4) incomplete medical records.

This multicenter clinical study was approved by the Ethics Committee of Beijing Ditan Hospital (Approval No. Jing Di Lun Ke Zi [2018] (027)-03) and conducted in accordance with the ethical principles of the Declaration of Helsinki. As the lcoordinating center, Beijing Ditan Hospital oversaw the study protocol and submitted the approved documentation to the ethics committees of all participating centers for registration. Written informed consent was obtained from both patients and their legal guardians prior to study enrollment. The trial is registered with the Chinese Clinical Trial Registry (ChiCTR1900023284).

### 2.3 Clinical diagnosis and data collection

VE was defined as an acute alteration in mental status lasting ≥24 h at presentation without an alternative identifiable cause (Venkatesan et al., 2013). This definition additionally required either: (1) neuropathological findings consistent with encephalitis, or (2) acute microbiological or serological evidence of encephalitis from clinical specimens (Venkatesan et al., 2013). In cases of diagnostic uncertainty, consultation with neurology specialists was obtained.

The following data were systematically collected: demographic and epidemiological characteristics; clinical signs and symptoms; results of routine cerebrospinal fluid (CSF) analysis (including protein, glucose, and chloride levels); neuroimaging findings; length of hospital stay; in-hospital complications; and total hospitalization costs.

Adverse drug reactions were monitored and documented throughout hospitalization. Functional outcomes at discharge were assessed using the modified Rankin Scale (mRS). According to established criteria, an mRS score of 0–2 was considered a favorable prognosis, whereas a score of ≥3 indicated a poor prognosis (Saver et al., 2021). All patients were followed for 6 months after discharge, and detailed records of their sequelae were obtained. All data were collected by trained personnel using standardized case report forms and subsequently uploaded to a secure, cloud-based electronic database for centralized management and analysis.

### 2.4 Treatment

All patients diagnosed with VE received treatment in accordance with established clinical guidelines (Tunkel et al., 2008). Based on neurologist recommendations, empirical antiviral therapy with intravenous acyclovir was administered at a dose of 10 mg/kg every 8 h. Mannitol was given intravenously at 1 g/kg every 8 h to reduce intracranial pressure. Supportive care, including maintenance of fluid and electrolyte balance and regulation of body temperature, was provided as needed. Patients in the XYPI group additionally received XYPI intravenously at a dose of 5–10 mg/kg once daily, with a maximum daily dose not exceeding 250 mg.

### 2.5 Statistical methods

SPSS 25 (IBM Corp., Armonk, NY, United States of America) and R software version 4.4.1 (R Foundation for Statistical Computing, Vienna, Austria) were used for analysis. The normality of continuous variables was assessed prior to analysis. Variables with a normal distribution were compared using Student’s t-test, whereas non-normally distributed variables were analyzed using the Mann–Whitney U test. Categorical variables were compared using the Chi-square test or Fisher’s exact test, as appropriate. Data are expressed as mean ± standard deviation for normally distributed variables, median (25th–75th percentile) for non-normally distributed variables, and number (%) for categorical variables. To reduce selection bias and estimate the unbiased treatment effect between patients who received XYPI and those who did not, propensity score matching (PSM) was applied. Univariate and multivariate logistic regression analyses were used to calculate odds ratios (ORs) and 95% confidence intervals (CIs) for poor prognosis in patients with VE. The cumulative incidence of poor prognosis was estimated using Kaplan–Meier survival curves, and group differences were evaluated with the log-rank test. P value <0.05 was considered statistically significant.

## 3 Results

### 3.1 Baseline characteristics

**FIGURE 1:**
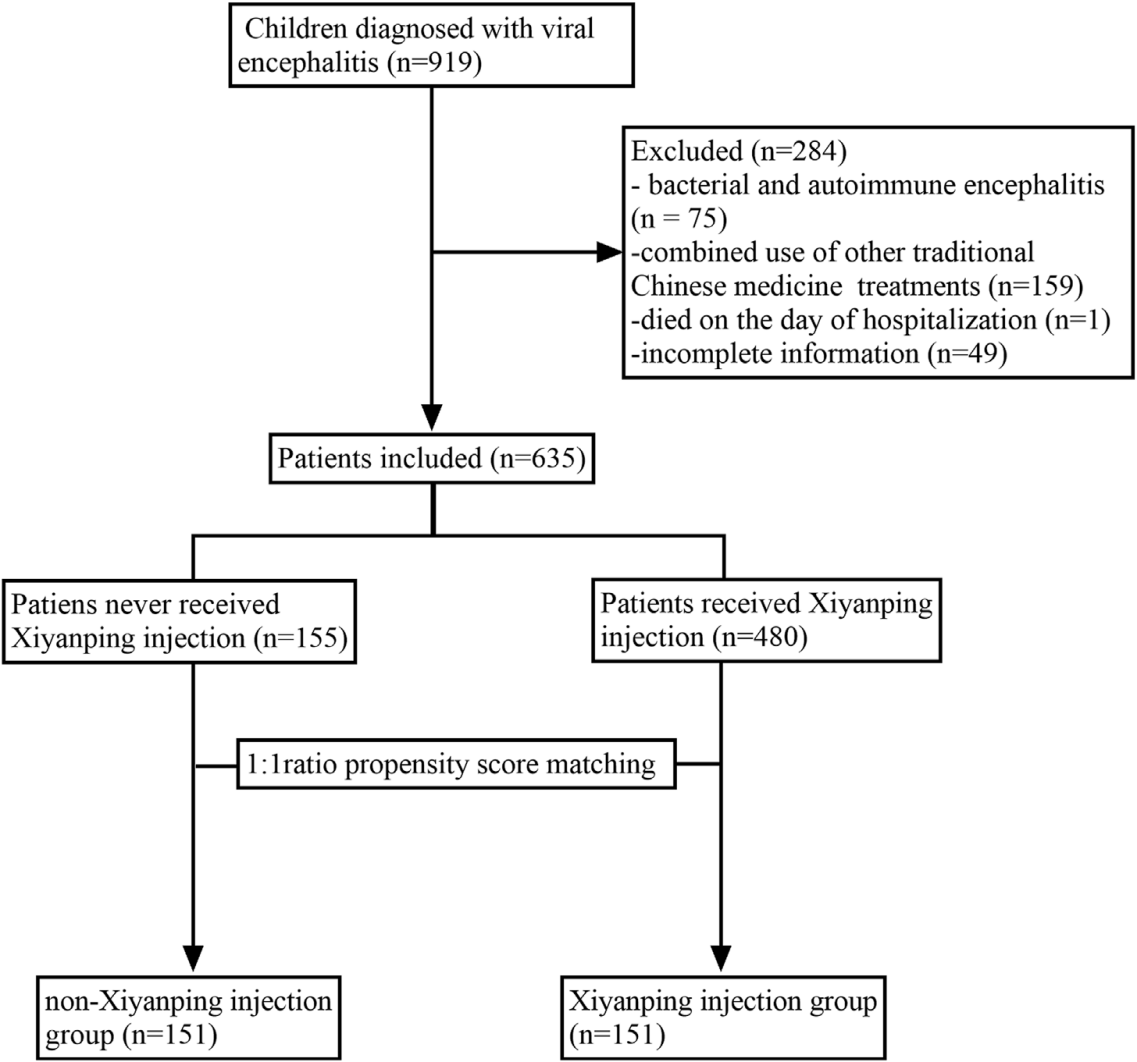
Flow chart of patient enrollment and selection for pediatric viral encephalitis.

A total of 635 inpatients were included in the final analysis and classified into the XYPI group (n = 480) or the non-XYPI group (n = 155), as shown in Figure 1. Before PSM, significant differences in baseline characteristics were observed between the two groups. Specifically, the XYPI group exhibited a higher prevalence of headache, whereas the non-XYPI group showed a significantly higher incidence of vertigo, fatigue, convulsions, and respiratory symptoms. CSF analysis revealed that glucose and chloride levels were higher in the non-XYPI group than in the XYPI group. After 1:1 PSM, the baseline clinical characteristics were well balanced between the two groups, as summarized in Table 1.

**TABLE 1:** Clinical characteristics of pediatric viral encephalitis in unmatched and propensity score–matched cohorts.

Variables	Unmatched cohort			Matched cohort		
	Non-Xiyanping injection user (n = 155)	Xiyanping injection user (n = 480)	P value	Non-Xiyanping injection user (n = 151)	Xiyanping injection user (n = 151)	P value
Sex, male	103 (66.45%)	334 (69.58%)	0.464	101 (66.89%)	94 (62.25%)	0.400
Age, year	4.14 (2.05, 8.00)	4.99 (3.19, 7.29)	0.203	4.11 (2.05, 8.00)	4.99 (3.19, 7.29)	0.835
BMI	16.00 (14.58, 18.03)	16.02 (14.51, 18.34)	0.920	15.87 (14.51, 18.00)	16.25 (14.60, 18.75)	0.436
Family history	4 (2.58%)	3 (0.63%)	0.064	4 (2.65%)	1 (0.66%)	0.371
Prehospital care	79 (50.97%)	229 (47.71%)	0.480	81 (53.64%)	72 (47.68%)	0.300
Admission temperature, °C	37.50 (36.90, 38.10)	37.45 (36.90, 38.00)	0.321	37.50 (36.90, 38.20)	37.40 (36.80, 37.90)	0.150
GCS	13.00 (13.00, 13.00)	13.00 (10.00, 15.00)	0.054	13.00 (13.00, 13.00)	13.00 (10.00, 15.00)	0.083
Meningeal irritation sign	7 (4.52%)	14 (2.92%)	0.326	5 (3.31%)	2 (1.32%)	0.448
Marie ataxia	2 (1.29%)	0 (0.00%)	0.059	2 (1.32%)	0 (0.00%)	0.498
pathologic reflex	27 (17.42%)	82 (17.08%)	0.898	25 (16.56%)	23 (15.23%)	0.875
Headache	64 (41.29%)	359 (74.79%)	<0.001	63 (41.72%)	73 (48.34%)	0.247
Vertigo	17 (10.97%)	17 (3.54%)	<0.001	14 (9.27%)	9 (5.96%)	0.278
Unconsciousness	7 (4.52%)	21 (4.38%)	0.941	6 (3.97%)	5 (3.31%)	0.759
Convulsions	36 (23.23%)	75 (15.63%)	0.030	35 (23.18%)	25 (16.56%)	0.150
Thirst	17 (10.97%)	58 (12.08%)	0.708	16 (10.60%)	17 (11.26%)	0.854
Fatigue	31 (20.00%)	59 (12.29%)	0.026	27 (17.88%)	20 (13.25%)	0.256
Digestive symptoms	37 (23.87%)	138 (28.75%)	0.237	41 (27.15%)	37 (24.50%)	0.599
Respiratory symptoms	21 (13.55%)	64 (13.33%)	0.007	20 (13.25%)	18 (11.92%)	0.729
CAR	0.06 (0.032, 0.08)	0.06 (0.04, 0.10)	0.312	0.06 (0.03, 0.09)	0.06 (0.04, 0.08)	0.945
CSF nucleated cells (10^6^/L)	99.0. (30.00, 166.50)	86.00 (36.00, 160.00)	0.569	99.5 (30.00, 170.00)	80.00 (30.00, 170.00)	0.864
CSF protein (g/L)	0.22 (0.15, 0.32)	0.20 (0.16, 0.29)	0.267	0.25 (0.16, 0.38)	0.20 (0.15, 0.30)	0.197
CSF glucose (mmol/L)	3.47 (3.13, 3.85)	3.33 (3.03, 0.73)	0.040	3.41 (3.06, 3.72)	3.36 (3.07, 3.73)	0.114
CSF chloride (mmol/L)	124.00 (120.98, 126.00)	122.00 (119.78, 125.00)	0.003	123.60 (120.00, 125.00)	123.00 (120.00, 125.00)	0.139
Abnormal EEG	26 (16.77%)	85 (17.71%)	0.949	25 (16.56%)	25 (16.56%)	0.930
Abnormal brain CT or MRI	21 (13.55%)	18 (3.75%)	0.906	20 (13.25%)	4 (2.65%)	0.437

BMI, body mass index; GCS, glasgow coma scale; CAR, C-reactive protein-to-albumin ratio; CSF, cerebrospinal fluid; EEG, electroencephalogram.

### 3.2 Prognostic risk factors

As shown in Table 2, multivariate logistic regression analysis demonstrated that, even after adjusting for potential confounders—including family history, prehospital care, headache, vertigo, convulsions, fatigue, and GCS score—XYPI use was independently associated with a significantly reduced risk of poor prognosis in patients with VE (OR = 0.251, 95% CI: 0.113–0.559, p = 0.001). This analysis also identified vertigo (OR = 4.447, 95% CI: 1.476–13.400, p = 0.009) and fatigue (OR = 2.923, 95% CI: 1.203–7.104, p = 0.006) as significant independent risk factors for poor outcomes, whereas headache (OR = 0.302, 95% CI: 0.127–0.715, p = 0.006) and higher GCS scores (OR = 0.677, 95% CI: 0.552–0.830, p < 0.001) were protective factors.

**TABLE 2:** Multivariate analysis of risk factors for poor prognosis in patients with viral encephalitis after propensity score matching.

Variables	Univariate analysis		Multivariate analysis	
	OR 95% CI	P values	OR 95% CI	P values
Xiyanping use	0.366 (0.190, 0.703)	0.003	0.251 (0.113, 0.559)	0.001
Family history	7.979 (1.298, 49.053)	0.025	2.236 (0.281, 17.785)	0.447
Prehospital care	1.925 (1.027, 3.607)	0.041	1.797 (0.872, 3.701)	0.112
Admission temperature, °C	1.257 (0.955, 1.656)	0.103		
Meningeal irritation sign	0.837 (0.099, 7.105)	0.870		
Marie ataxia	5.122 (0.315, 83.287)	0.251		
Pathologic reflex	0.838 (0.352, 1.992)	0.689		
Headache	0.326 (0.163, 0.653)	0.002	0.302 (0.127, 0.715)	0.006
Vertigo	3.732 (1.516, 9.184)	0.004	4.447 (1.476, 13.400)	0.009
Unconsciousness	3.043 (0.856, 10.818)	0.085		
Convulsions	2.812 (1.445, 5.473)	0.002	0.873 (0.148, 0.516)	0.881
Thirst	0.669 (0.224, 1.994)	0.470		
Fatigue	2.569 (1.253, 5.266)	0.010	2.923 (1.203, 7.104)	0.038
Digestive symptoms	0.676 (0.321, 1.427)	0.305		
Respiratory symptoms	1.161 (0.480, 2.806)	0.741		
GCS	0.761 (0.655, 0.884)	<0.001	0.677 (0.552, 0.830)	<0.001
CSF nucleated cells (10^6^/L)	1.000 (0.998, 1.003)	0.717		
CSF protein (g/L)	0.563 (0.058, 5.497)	0.621		
CSF glucose (mmol/L)	1.641 (0.933, 2.883)	0.085		
CSF chloride (mmol/L)	1.029 (0.928, 1.142)	0.583		
Abnormal EEG	1.442 (0.746, 2.789)	0.277		
Abnormal brain CT or MRI	1.170 (0.504, 2.715)	0.714		

GCS, glasgow coma scale; CSF, cerebrospinal fluid; EEG, electroencephalogram.

Additionally, a separate multivariate logistic regression analysis of all patients at baseline, adjusting for family history, prehospital care, headache, vertigo, convulsions, fatigue, dgestive symptoms, respiratory symptoms, and GCS score, confirmed that XYPI use remained a significant protective factor against poor prognosis (OR = 0.409, 95% CI: 0.170–0.987, p = 0.047). The detailed results of this analysis are provided in Supplementary Table S1.

### 3.3 Incidence of poor prognosis

**FIGURE 2:**
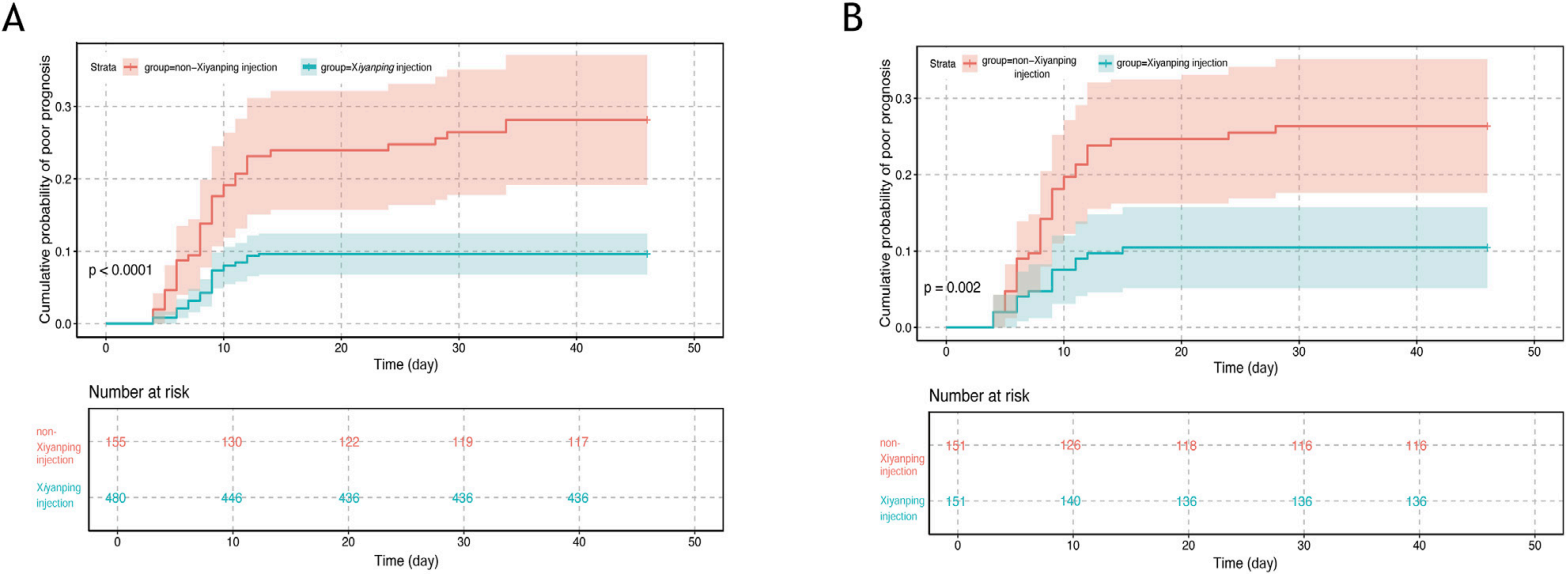
Cumulative incidence of poor prognosis in pediatric viral encephalitis. **(A)** Unmatched cohort: cumulative incidence of poor prognosis in patients receiving Xiyanping injection *versus* non-Xiyanping injection treatment (9.17% vs. 24.51%; log-rank test, P < 0.0001). **(B)** Propensity score–matched cohort: cumulative incidence of poor prognosis in Xiyanping injection *versus* non-Xiyanping injection groups (9.93% vs. 23.17%; log-rank test, P = 0.002).

Comprehensive analysis revealed that the incidence of poor prognosis was significantly lower among patients receiving XYPI during hospitalization (9.17%) compared with those in the non-XYPI group (Figure 2A). Consistently, PSM analysis demonstrated that the XYPI group exhibited a markedly lower incidence of poor outcomes (9.93%) than the matched non-XYPI group (23.17%, Figure 2B).

### 3.4 Symptoms, financial burden, and comorbidities

**FIGURE 3:**
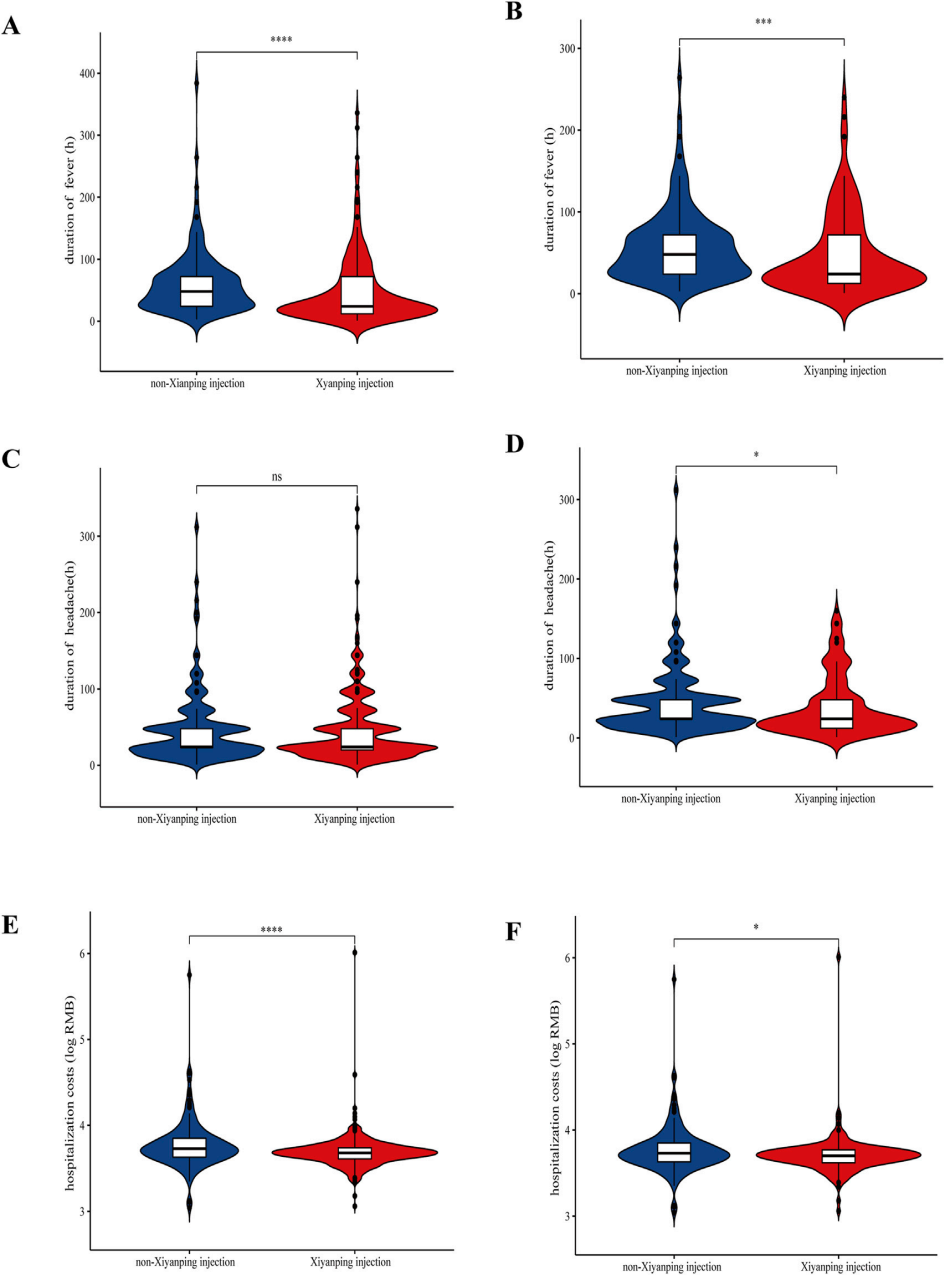
Violin plots illustrating the effect of Xiyanping injection on symptom duration and hospitalization costs. **(A)** Duration of fever (unmatched patients); **(B)** duration of fever (matched patients); **(C)** duration of headache (unmatched patients); **(D)** duration of headache (matched patients); **(E)** hospitalization costs (unmatched patients); **(F)** hospitalization costs (matched patients). P values: *p < 0.05, **p < 0.01, ***p < 0.001.

A comprehensive analysis was conducted to evaluate the effects of XYPI treatment on clinical symptoms, economic outcomes, and in-hospital complications. XYPI administration significantly alleviated fever (Figures 3A,B). Although headache duration showed no baseline difference between groups (Figure 3C), XYPI treatment significantly shortened headache duration after matching (Figure 3D).

Moreover, XYPI therapy was associated with a significant reduction in hospitalization costs. Before matching, the mean cost for patients not receiving XYPI was 1.12-fold higher than that for XYPI-treated patients. After matching, hospitalization costs in the non-XYPI group remained significantly higher than those in the XYPI group (Figures 3E,F; Supplementary Table S2).

All in-hospital complications were carefully recorded. XYPI administration was associated with a lower incidence of respiratory tract infections (Supplementary Figures S1A, S1B) and a reduced risk of myocardial injury (Supplementary Figures S1C, S1D). In contrast, the frequencies of gastrointestinal dysfunction (Supplementary Figures S1E, S1F) and sepsis (Supplementary Figures S1G, S1H) did not differ significantly between groups.

### 3.5 Drug-related adverse reactions and sequelae

Drug-related adverse events were systematically monitored and recorded in all hospitalized patients, with particular attention to diarrhea, rash, and chills. Compared with patients who did not receive XYPI, those treated with XYPI experienced a significantly lower incidence of chills (Supplementary Figures S2A, S2B) and rash (Supplementary Figures S2C, S2D), both before and after PSM. In contrast, the incidence of diarrhea did not differ significantly between the two groups (Supplementary Figures S2E, S2F).

At the 6-month follow-up, the majority of patients demonstrated favorable recovery after discharge. However, a small subset exhibited residual sequelae, including gait disturbance (n = 3), intellectual disability (n = 3), and epileptic symptoms (n = 2). Notably, the incidence of sequelae was significantly higher in the non-XYPI group compared with the XYPI group (Supplementary Table S3).

## 4 Discussion

VE is a life-threatening neurological disorder that can lead to death or long-term disability, posing a serious threat to the healthy development of children and underscoring the urgent need for effective and well-tolerated therapeutic strategies. A recent prospective study reported that, after a 2-year follow-up, approximately 67% of patients with VE continued to experience cognitive impairment, fatigue, and other debilitating sequelae, regardless of initial disease severity, highlighting the persistent and pervasive impact of VE on long-term health outcomes (Schwitter et al., 2024).

At present, the treatment of VE remains largely empirical, relying mainly on antimicrobial therapy and supportive care. Although these approaches constitute the standard of care, growing evidence indicates that TCM may offer beneficial adjunctive effects in pediatric VE, highlighting the potential value of developing integrative treatment strategies that combine it with conventional medicine (Tunkel et al., 2008; Wang et al., 2024).

The mRS is a comprehensive and objective measure of functional disability, providing a standardized framework that minimizes subjective bias. Widely used in various neurological disorders, including acute stroke and cerebral small vessel disease, it enables clinicians to reliably assess and monitor functional status and disability levels (Hosoya et al., 2024; Clancy et al., 2024). In this study, patients’ prognoses were systematically evaluated and stratified using the mRS. Our findings demonstrated that XYPI administration was an independent protective factor against poor outcomes, significantly reducing the risk of unfavorable prognosis.

The GCS, one of the earliest tools developed to evaluate impaired consciousness, coma depth, and duration, encompasses three core components: motor responsiveness, verbal performance, and eye opening (Teasdale and Jennett, 1974). It remains one of the most universally adopted instruments for assessing the level of consciousness and is widely applied in neurological disorders such as acute intracranial injury and stroke (Easter et al., 2015). Consistent with previous reports, our study revealed a significant association between GCS scores and prognostic outcomes, demonstrating that lower GCS scores are predictive of poor prognosis in pediatric encephalitis (Feng et al., 2020; Rebecca et al., 2024; Ngan et al., 2024).

Our study demonstrated that XYPI effectively alleviates clinical symptoms and improves patient prognosis, an effect likely attributable to its multifaceted pharmacological activities, including anti-inflammatory, antiviral, and neuroregulatory properties. Previous studies have shown that AS, the principal active component of XYPI, not only directly inhibits adenovirus replication but also enhances host immunity by modulating neutrophil and T-cell function and promoting macrophage phagocytic activity. These immunomodulatory actions facilitate viral clearance and the release of antiviral factors, thereby suppressing viral replication (Zhang J et al., 2021; Li et al., 2025). Moreover, XYPI mitigates cytokine storm through four key mechanisms: inhibition of p38 phosphorylation, suppression of MAPK phosphorylation, reduction of NF-κB phosphorylation, and negative regulation of STAT3 activation (Li et al., 2025).

The neuroprotective potential of XYPI has been demonstrated across diverse preclinical models, confirming its target-organ specificity for the central nervous system. Permeability–surface area product analyses by Zhou et al. revealed that XYPI stabilizes the blood–brain barrier by modulating paracellular transport and attenuating neuroinflammatory cascades (Zhou et al., 2024). Emerging evidence further highlights the dual therapeutic efficacy of XYPI in promoting neurological recovery and exerting neuro-oncological effects, particularly through the modulation of glioma stem cell plasticity (Zhang L et al., 2021; Othman and Mohd, 2022).

Our study also identified a notable finding: the duration of fever was significantly longer in patients who did not receive XYPI. This observation is consistent with the report by Yang et al., which demonstrated that prolonged fever is a risk factor for poor prognosis in VE (Yang et al., 2024). The antipyretic effect of XYPI may be explained by its ability to suppress the release of pyro-inflammatory mediators such as interleukin-1 and tumor necrosis factor-α, while simultaneously reducing the production of positive regulatory factors, including prostaglandin E2 and 5-hydroxytryptamine, within the hypothalamic fever center (Liu et al., 2014; Guo et al., 2012; Peng et al., 2016a; Peng et al., 2016b). By modulating these key mediators, XYPI may attenuate the febrile response, thereby shortening fever duration and potentially improving clinical outcomes in VE.

The precise causal relationships between VE and the development of complex systemic infections, respiratory diseases, and gastrointestinal disorders warrant further investigation (Bohmwald et al., 2018). Notably, our results demonstrated that XYPI significantly reduced the incidence of respiratory tract infections, an effect that may be attributable to its capacity to ameliorate pathological lung changes, alleviate pulmonary edema, and suppress inflammatory cytokine production (Peng et al., 2016a; Christie et al., 2007; Glaser and Bloch, 2020). Moreover, XYPI administration was associated with a lower rate of myocardial injury. Supporting this finding, Zhang et al. reported the efficacy of XYPI in children with myocardial damage based on real-world data from 39 hospitals across China (Zhang X. Y et al., 2021). Preclinical studies further suggest that these cardioprotective effects may involve activation of the peroxisome proliferator-activated receptor-α signaling pathway (Zhang et al., 2024).

VE imposes a substantial economic burden worldwide. Wang et al. reported that encephalitis caused by Chinese flavivirus in children continues to exhibit a high prevalence of severe cases, particularly in rural regions (Wang et al., 2022). Similarly, Tarantola et al. demonstrated that in the Mekong region—a hotspot for VE—the financial burden associated with this disease exceeds the average income of the general population by nearly tenfol (Tarantola et al., 2014). In the United States, the average cost of encephalitis treatment doubled between 1998 and 2010 (Vora et al., 2014). Consistent with these observations, our findings further highlighted the potential of XYPI to alleviate the economic burden of inpatient care.

Although the incidence of sequelae was relatively low in our cohort, VE remains a life-threatening condition in children. Viruses can invade the central nervous system via three primary routes: peripheral nerves connecting to the brain, olfactory neurons within the nasal epithelium, or direct penetration across the blood–brain barrierr (Sun et al., 2024). Such infections can cause irreversible neural injury, resulting in long-lasting neurological sequelae. Notably, XYPI has been reported to reduce the risk of virus-related sequelae, potentially by inhibiting hemagglutinin adsorption and enhancing immune cell activity, thereby strengthening host antiviral defenses (Wen et al., 2015).

The present study, although retrospective in design, provides valuable evidence supporting the potential therapeutic benefits of XYPI in mitigating VE-related sequelae. Nevertheless, high-quality prospective trials are warranted to conclusively establish its efficacy. In addition, the absence of adult VE data limits the generalizability of our findings. Another limitation is the lack of proteomic profiling and dynamic cytokine analyses of cerebrospinal fluid, which precludes a deeper understanding of the mechanisms underlying XYPI’s effects in pediatric VE. Future studies incorporating these molecular approaches are needed to validate the current findings and elucidate the pathways mediating XYPI’s therapeutic action.

## 5 Conclusion

This study demonstrates that XYPI effectively reduces adverse outcomes, alleviates clinical symptoms, and lessens the economic burden in pediatric patients with VE. However, larger prospective, multicenter trials are warranted to definitively confirm its efficacy and safety.

## Data Availability

The original contributions presented in the study are included in the article/Supplementary Material, further inquiries can be directed to the corresponding authors.
