# Supplementary material for: Effect of adjuvant treatment with Xiyanping injection on the prognosis of viral encephalitis in children: a multicenter retrospective study
Source: Front Pharmacol. 2025 Oct 30;16:1632728. doi: 10.3389/fphar.2025.1632728 (PMC12611970; doi:10.3389/fphar.2025.1632728)
Supplement: Supplementary file 1 [file Table1.docx]

Table S1 Multivariate analysis of risk factors for poor prognosis in patients with viral encephalitis before propensity score matching.

| Variables | Univariate Analysis | | Multivariate Analysis | |
| --- | --- | --- | --- | --- |
|  | OR 95% CI | P values | OR 95% CI | P values |
| Xiyanping use | 0.334(0.208,0.537) | <0.001 | 0.409(0.170,0.987) | 0.047 |
| Family history | 4.997(1.098,22.716) | 0.037 | 0.000(0.000,0.000) | 0.999 |
| Prehospital care | 1.167(1.107,2.570) | 0.042 | 0.895(0.447,1.790) | 0.753 |
| Meningeal irritation sign | 1.545(0.507,4.708) | 0.444 |  |  |
| Marie ataxia | 6.524(0.404,105.295) | 0.186 |  |  |
| Pathologic reflex | 0.766(0.400,1.465) | 0.421 |  |  |
| Headache | 0.347(0.218,0.552) | <0.001 | 0.630(0.302,1.312) | 0.217 |
| Vertigo | 0.241(0.114,0.511) | <0.001 | 3.042(1.051,8.806) | 0.040 |
| Unconsciousness | 1.432(0.529,3.874) | 0.479 |  |  |
| Convulsions | 2.411(1.439,4.0339) | 0.001 | 0.331(0.030,3.709) | 0.370 |
| Thirst | 0.749(0.346,1.620) | 0.463 |  |  |
| Fatigue | 3.922(2.320,6.628) | <0.001 | 1.510(0.609,3.747) | 0.374 |
| Digestive symptoms | 0.575(0.357,0.925) | 0.022 | 0.871(0.392,1.934) | 0.734 |
| Respiratory symptoms | 2.783(1.534,5.048) | 0.001 | 2.741(1.269,5.922) | 0.010 |
| GCS | 0.705(0.621,0.800) | <0.001 | 0.578(0.475,0.704) | <0.001 |
| CSF nucleated cells (10^6^/l) | 1.001(0.999,1.002) | 0.450 |  |  |
| CSF protein (**g/L)** | 2.016(0.876,4.640) | 0.099 |  |  |
| CSF glucose (**mmol/L)** | 1.330(0.836,2.116) | 0.229 |  |  |
| CSF chloride (**mmol/L)** | 1.026(0.955,1.104) | 0.482 |  |  |
| Abnormal EEG | 1.933(0.932,4.012) | 0.077 |  |  |
| Abnormal brain CT or MRI | 1.868(0.868,4.017) | 0.110 |  |  |

GCS, Glasgow Coma Scale; CSF, Cerebrospinal fluid; EEG, Electroencephalogram
