# Supplementary figures and images for: Effect of adjuvant treatment with Xiyanping injection on the prognosis of viral encephalitis in children: a multicenter retrospective study
Source: Front Pharmacol. 2025 Oct 30;16:1632728. doi: 10.3389/fphar.2025.1632728 (PMC12611970; doi:10.3389/fphar.2025.1632728)

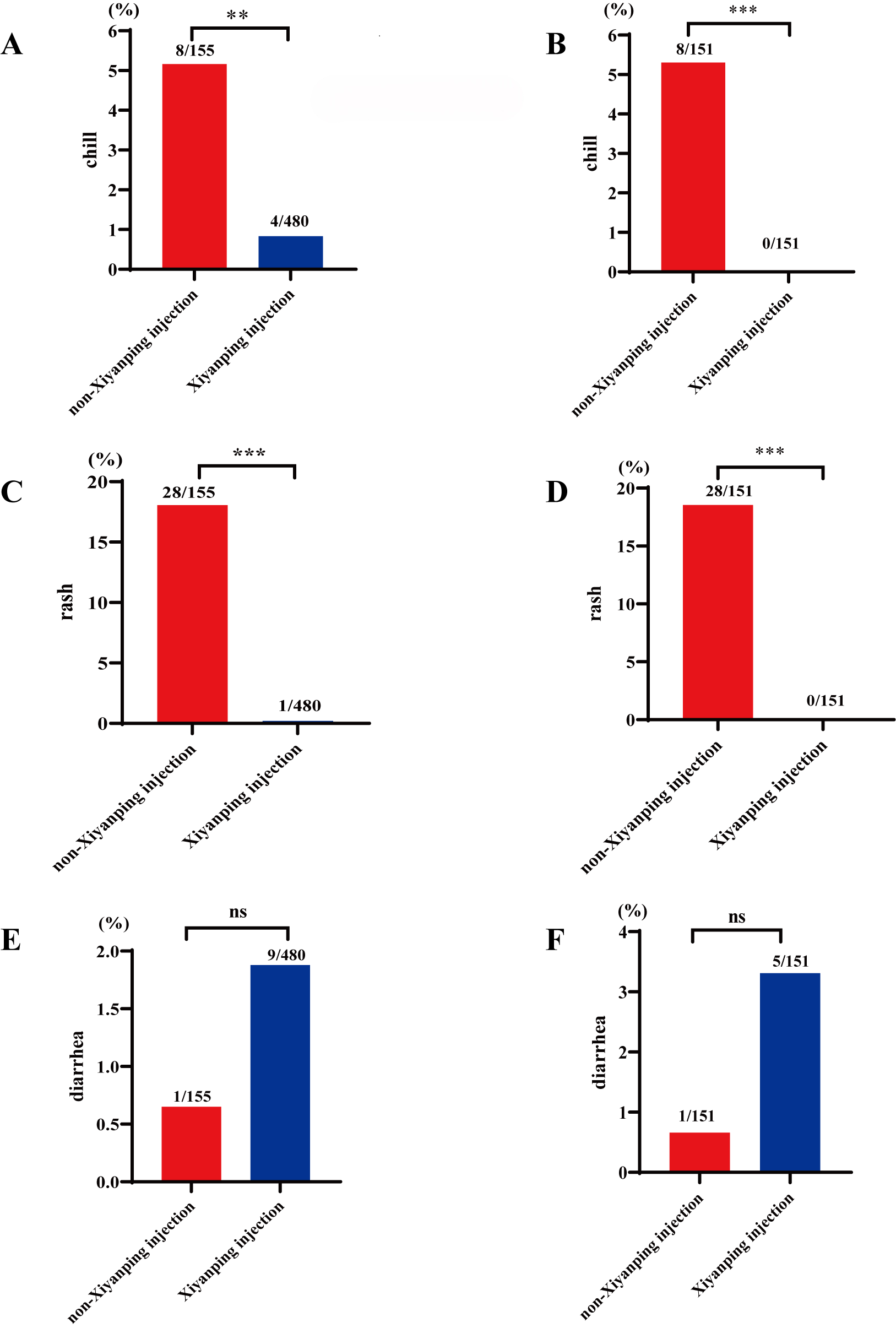

Supplement: Supplementary file 2 [file Image2.tif]

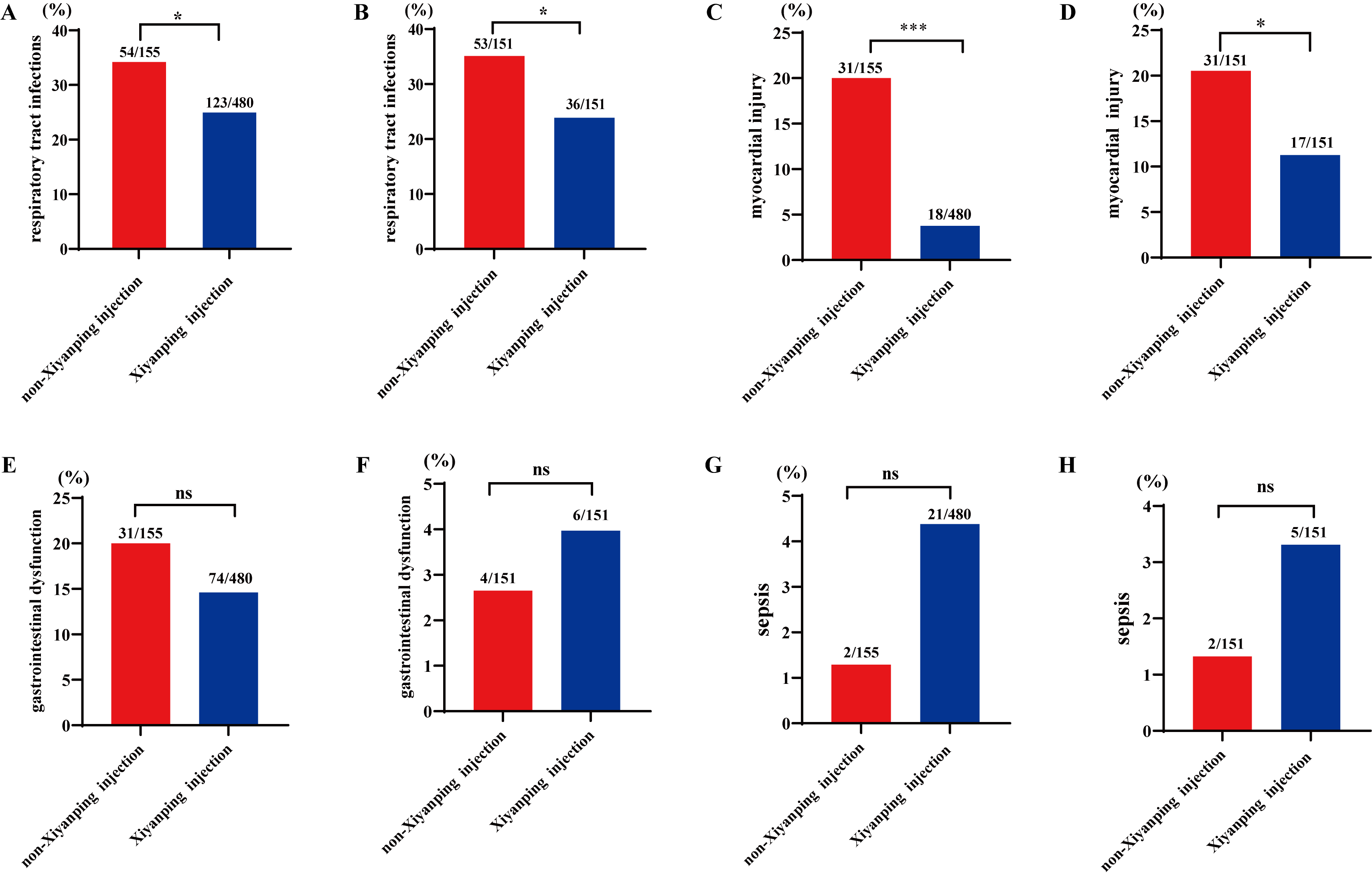

Supplement: Supplementary file 3 [file Image1.tif]
