# Supplementary material for: Effect of adjuvant treatment with Xiyanping injection on the prognosis of viral encephalitis in children: a multicenter retrospective study
Source: Front Pharmacol. 2025 Oct 30;16:1632728. doi: 10.3389/fphar.2025.1632728 (PMC12611970; doi:10.3389/fphar.2025.1632728)
Supplement: Supplementary file 4 [file Table2.docx]

Table S2 Comparison of hospitalization costs between the Xiyanping and non-Xiyanping injection groups in unmatched and matched cohorts.

| Variables | Unmatched cohort | | | Matched cohort | | |
| --- | --- | --- | --- | --- | --- | --- |
|  | Non-Xiyanping injection user (n=155) | Xiyanping injection user (n=480) | P value | Non-Xiyanping injection user （n=151） | Xiyanping injection user（n=151） | P value |
| Hospitalization cost ( yuan) | 5400.35(4226.85,7146.12) | 4821.90(4116.73,5544.93) | <0.001 | 5329.35(4168.61,5857.90) | 4947.45（4168.61,5857.90） | 0.025 |
