# Supplementary material for: Effect of adjuvant treatment with Xiyanping injection on the prognosis of viral encephalitis in children: a multicenter retrospective study
Source: Front Pharmacol. 2025 Oct 30;16:1632728. doi: 10.3389/fphar.2025.1632728 (PMC12611970; doi:10.3389/fphar.2025.1632728)
Supplement: Supplementary file 5 [file DataSheet1.pdf]

J13192

江西青峰药业有限公司
产品检验报告书

报告书编号：CZ12201904007　　　　文件编号：R-SMP-QA-1011-032-02-A0

产品名称	喜炎平注射液	批　　号	2019041803
规　　格	每支装2ml:50mg	来　　源	小容量注射剂车间(B线)
生产单位	江西青峰药业有限公司	生产日期	2019年04月18日
包　　装	安瓿	有 效 期	至2020年09月
检验项目	全检	报告日期	2019年05月16日
检验依据	国家食品药品监督管理局标准国家药品标准WS-10863(ZD-0863)-2002-2011Z、《中国药典》2015年版四部通则、企业标准（TS-QA-1011-CCP0100101-A5）		

检验项目	标准规定	检验结果
【性状】	应为淡黄色至黄绿色的澄明液体	为黄绿色的澄明液体
【鉴别】		
(1)化学反应	应呈正反应	呈正反应
(2)化学反应	应呈正反应	呈正反应
(3)薄层色谱	应在与17-氢-9-去氢穿心莲内酯-19-硫酸酯钠对照品色谱相应的位置上，显相同颜色的斑点	在与17-氢-9-去氢穿心莲内酯-19-硫酸酯钠对照品色谱相应的位置上，显相同颜色的斑点
【检查】		
溶液的颜色	应符合规定	符合规定
pH值	应为4.5～6.5	5.8
蛋白质	应符合规定	符合规定
鞣质	应符合规定	符合规定
草酸盐	应符合规定	符合规定
钾离子	应符合规定	符合规定
树脂	应符合规定	符合规定
重金属	应不得过百万分之十	符合规定
砷盐	应不得过百万分之二	符合规定
炽灼残渣	应不得过1.5%(g/ml)	0.9%
重金属及有害元素残留量	按本品每日最大使用量计算，铅不得超过12μg，镉不得超过3μg，砷不得超过6μg，汞不得超过2μg，铜不得超过150μg	铅：0.1μg，镉：0.004μg，砷：0.03μg，汞：0.06μg，铜：0.1μg
装量	应符合规定	符合规定
可见异物	应符合规定	符合规定
不溶性微粒	应符合规定	符合规定
溶血与凝聚	应符合规定	符合规定
热原	应符合规定	符合规定
大分子物质	应不得检出分子量大于生长抑素的大分子物质	未检出
异常毒性	应符合规定	符合规定
过敏反应	应符合规定	符合规定
无菌	应符合规定	符合规定
细菌内毒素	每1ml中含细菌内毒素的量应小于0.50EU	符合规定
【指纹图谱】	应分别呈现与参照物色谱峰保留时间相同的色谱峰；与对照指纹图谱的相似度不得低于0.85	分别呈现与参照物色谱峰保留时间相同的色谱峰；相似度为0.97
【含量测定】		
总磺化物	本品每1ml含穿心莲内酯总磺化物以穿心莲内酯磺酸钠($C_{20}H_{29}O_5 \cdot SO_3Na$)计，应为标示量的85.0%～115.0%	96.6%
17-氢-9-去氢穿心莲内酯-19-硫酸酯钠	本品每1ml含穿心莲内酯总磺化物以17-氢-9-去氢穿心莲内酯-19-硫酸酯钠($C_{20}H_{29}O_5 \cdot SO_3Na$)计，应不得少于0.50mg	0.92mg

结论：本品按国家食品药品监督管理局标准国家药品标准WS-10863(ZD-0863)-2002-2011Z、《中国药典》2015年版四部通则、企业标准（TS-QA-1011-CCP0100101-A5）检验，结果符合规定。

审核人：黄岩芳　　　　QC：[illegible]

江西青峰药业有限公司 质量保证部

江西青峰医药贸易有限公司 质量管理部

JiangxiQingfeng Pharmaceutical Co.,Ltd.

Product Inspection Report

Report Number：CZ12201904007 Document Number：R-SMP-QA-1011-032-02-A0

Product Name	Xiyanping_injection	Batch Number	2019041803
Specifications	Each vial contains 2ml:50mg	Provenance	Small-Volume Parenteral (SVP) Workshop (Line B)
Manufacturer	Jiangxi Qingfeng Pharmaceutical Co.,	Production Date	April 18, 2019
Packaging	ampoule	Expiration Date	September 2020
Inspection Items	Full inspection	Report Date	May 16, 2019
Basis for Inspection	National Medical Products Administration (NMPA) Standard: National Drug Standard (3410863(Z0-0863)-2002-2011Z), Chinese Pharmacopoeia 2015 Edition, General Chapters Volume IV, and Enterprise Standard TS-06-1010070100101-A5		

**Inspection Items**	**Standard Requirements**	**Inspection Results**
**[Description]**	It should be a clear, light yellow to yellow-green liquid.	A clear, yellow liquid.
**[Identification]**		
(1) Chemical reaction	shall give a positive reaction	positive reaction.
(2) Chemical reaction	shall give a positive reaction	positive reaction.
(3)Thin-layer chromatography	shall show a spot of the same color in the position corresponding to that of the 17-Hydroxy-9-dehydroandrographolide-19-sulfate Sodium reference standard.	The chromatogram shall show a spot of the same color in the position corresponding to that of the17-Hydroxy-9-dehydroandrographolide-19-sulfate Sodium reference standard.
**[Tests]**		
Solution Color	Shall comply with the specifications	Conforms to the requirements
pH Value	The value should be between 4.5 and 6.5	5.8
Protein	Shall comply with the specifications	Conforms to the requirements
Tannins	Shall comply with the specifications	Conforms to the requirements
Oxalates	Shall comply with the specifications	Conforms to the requirements
Potassium Ions	Shall comply with the specifications	Conforms to the requirements
Resins	Shall comply with the specifications	Conforms to the requirements
Heavy Metals	Shall not exceed ten parts per million (10 ppm)	Conforms to the requirements
Arsenic Salt	Shall not exceed two parts per million (2 ppm)	Conforms to the requirements

Residue on Ignition	should not exceed 1.5% (g/mL).	0.9%
Residual Heavy Metals and Harmful Elements	Based on the maximum daily intake of this product, the limits shall not exceed: 12 μg for lead (Pb), 3 μg for cadmium (Cd), 6 μg for arsenic (As), 2 μg for mercury (Hg), and 150 μg for copper (Cu)	Lead (Pb): 0.14 μg; Cadmium (Cd): 0.004 μg; Arsenic (As): 0.034 μg; Mercury (Hg): 0.064 μg; Copper (Cu): 0.14 μg
Fill Volume	Shall comply with the specifications	Conforms to the requirements
Visible Particulates	Shall comply with the specifications	Conforms to the requirements
Insoluble Particulates	Shall comply with the specifications	Conforms to the requirements
Hemolysis and Aggregation	Shall comply with the specifications	Conforms to the requirements
Pyrogens	Shall comply with the specifications	Conforms to the requirements
Macromolecular Substances	The high-molecular-weight substances with molecular weight greater than somatostatin should not be detectable.	Not Detected
Abnormal Toxicity	Shall comply with the specifications	Conforms to the requirements
Allergic Reaction	Shall comply with the specifications	Conforms to the requirements
Sterility	Shall comply with the specifications	Conforms to the requirements
Bacterial Endotoxins	The amount of bacterial endotoxins per 1 ml should be less than 0.50 EU.	Conforms to the requirements
Fingerprint	The test sample shall exhibit chromatographic peaks with retention times identical to those of the reference substance; and the similarity compared with the reference fingerprint chromatogram shall not be less than 0.85.	Chromatographic peaks corresponding to the reference substance with identical retention times were observed, exhibiting a similarity of 0.97.

[Assay]

Total Sulfonated Compounds	The content of total sulfonated andrographolides per 1 ml, calculated as andrographolide sulfonate sodium ($C_{20}H_{29}O_8S{\cdot}Na$), should be 85.0% to 115.0% of the labeled amount.	96.6%
17-Hydroxy-9-dehydro andrographolide-19-sulfate Sodium	The content of total sulfonated andrographolides per 1 ml, calculated as 17-hydro-9-dehydroandrographolide-19-sulfonate sodium ($C_{20}H_{27}O_8S{\cdot}Na$), shall be not less than 0.50 mg.	0.92mg

Conclusion: This product has been tested in compliance with the National Drug Standard WS-10863(20-0863)-2002-2011Z, the General Chapter 4 of the Chinese Pharmacopoeia 2015 Edition, and the Enterprise Standard TS-QA-1011-CCP0100101-A5, and the results meet all specified requirements.

Reviewer Qc:
