# Supplementary material for: Effect of adjuvant treatment with Xiyanping injection on the prognosis of viral encephalitis in children: a multicenter retrospective study
Source: Front Pharmacol. 2025 Oct 30;16:1632728. doi: 10.3389/fphar.2025.1632728 (PMC12611970; doi:10.3389/fphar.2025.1632728)
Supplement: Supplementary file 6 [file Table3.docx]

Table S3 Comparison of sequelae between the Xiyanping and non-Xiyanping injection groups in unmatched and matched cohorts.

| Variables | Unmatched cohort | | | Matched cohort | | |
| --- | --- | --- | --- | --- | --- | --- |
|  | non-Xiyanping injection user (n=155) | Xiyanping injection user (n=480) | P value | non-Xiyanping injection user （n=151） | Xiyanping injection user（n=151） | P value |
| Sequelae | 7(4.52%) | 1(0.21%) | <0.001 | 6(3.97%) | 0(0.00%) | 0.030 |
| Gait disturbance | 3(1.94%) | 0(0.00%) |  | 2(1.32%) | 0(0.00%) |  |
| Intellectual Disability | 3(1.94%) | 0(0.00%) |  | 3(1.97%) | 0(0.00%) |  |
| Epileptic symptoms | 1(0.65%) | 1(0.21%) |  | 1(0.66%) | 0(0.00%) |  |
