# Supplementary material for: Effect of adjuvant treatment with Xiyanping injection on the prognosis of viral encephalitis in children: a multicenter retrospective study
Source: Front Pharmacol. 2025 Oct 30;16:1632728. doi: 10.3389/fphar.2025.1632728 (PMC12611970; doi:10.3389/fphar.2025.1632728)
Supplement: Supplementary file 7 [file DataSheet2.docx]

Batch numbers of Xiyanping injection Product

| **Pharmaceutical product name** | **Specification(s)** | **Place of origin** | **Batch number** |
| --- | --- | --- | --- |
| Xiyanping_injection | 2ml:50mg | JiangxiQingfeng Pharmaceutical Co.,Ltd. | 2014102903 |
| Xiyanping_injection | 2ml:50mg | JiangxiQingfeng Pharmaceutical Co.,Ltd. | 2014102903 |
| Xiyanping_injection | 2ml:50mg | JiangxiQingfeng Pharmaceutical Co.,Ltd. | 2014102903 |
| Xiyanping_injection | 2ml:50mg | JiangxiQingfeng Pharmaceutical Co.,Ltd. | 2014102903 |
| Xiyanping_injection | 2ml:50mg | JiangxiQingfeng Pharmaceutical Co.,Ltd. | 2014121203 |
| Xiyanping_injection | 2ml:50mg | JiangxiQingfeng Pharmaceutical Co.,Ltd. | 2014121203 |
| Xiyanping_injection | 2ml:50mg | JiangxiQingfeng Pharmaceutical Co.,Ltd. | 2014090403 |
| Xiyanping_injection | 2ml:50mg | JiangxiQingfeng Pharmaceutical Co.,Ltd. | 2014121203 |
| Xiyanping_injection | 2ml:50mg | JiangxiQingfeng Pharmaceutical Co.,Ltd. | 2014121203 |
| Xiyanping_injection | 2ml:50mg | JiangxiQingfeng Pharmaceutical Co.,Ltd. | 2014121203 |
| Xiyanping_injection | 2ml:50mg | JiangxiQingfeng Pharmaceutical Co.,Ltd. | 2015012903 |
| Xiyanping_injection | 2ml:50mg | JiangxiQingfeng Pharmaceutical Co.,Ltd. | 2015012903 |
| Xiyanping_injection | 2ml:50mg | JiangxiQingfeng Pharmaceutical Co.,Ltd. | 2015012903 |
| Xiyanping_injection | 2ml:50mg | JiangxiQingfeng Pharmaceutical Co.,Ltd. | 2015020403 |
| Xiyanping_injection | 2ml:50mg | JiangxiQingfeng Pharmaceutical Co.,Ltd. | 2015020403 |
| Xiyanping_injection | 2ml:50mg | JiangxiQingfeng Pharmaceutical Co.,Ltd. | 2015020403 |
| Xiyanping_injection | 2ml:50mg | JiangxiQingfeng Pharmaceutical Co.,Ltd. | 2015030903 |
| Xiyanping_injection | 2ml:50mg | JiangxiQingfeng Pharmaceutical Co.,Ltd. | 2015030903 |
| Xiyanping_injection | 2ml:50mg | JiangxiQingfeng Pharmaceutical Co.,Ltd. | 2015030903 |
| Xiyanping_injection | 2ml:50mg | JiangxiQingfeng Pharmaceutical Co.,Ltd. | 2015030903 |
| Xiyanping_injection | 2ml:50mg | JiangxiQingfeng Pharmaceutical Co.,Ltd. | 2015041603 |
| Xiyanping_injection | 2ml:50mg | JiangxiQingfeng Pharmaceutical Co.,Ltd. | 2015041603 |
| Xiyanping_injection | 2ml:50mg | JiangxiQingfeng Pharmaceutical Co.,Ltd. | 2015041603 |
| Xiyanping_injection | 2ml:50mg | JiangxiQingfeng Pharmaceutical Co.,Ltd. | 2015051003 |
| Xiyanping_injection | 2ml:50mg | JiangxiQingfeng Pharmaceutical Co.,Ltd. | 2015060203 |
| Xiyanping_injection | 2ml:50mg | JiangxiQingfeng Pharmaceutical Co.,Ltd. | 2015060203 |
| Xiyanping_injection | 2ml:50mg | JiangxiQingfeng Pharmaceutical Co.,Ltd. | 2015060203 |
| Xiyanping_injection | 2ml:50mg | JiangxiQingfeng Pharmaceutical Co.,Ltd. | 2015060203 |
| Xiyanping_injection | 2ml:50mg | JiangxiQingfeng Pharmaceutical Co.,Ltd. | 2015060203 |
| Xiyanping_injection | 2ml:50mg | JiangxiQingfeng Pharmaceutical Co.,Ltd. | 2015060803 |
| Xiyanping_injection | 2ml:50mg | JiangxiQingfeng Pharmaceutical Co.,Ltd. | 2015060803 |
| Xiyanping_injection | 2ml:50mg | JiangxiQingfeng Pharmaceutical Co.,Ltd. | 2015060803 |
| Xiyanping_injection | 2ml:50mg | JiangxiQingfeng Pharmaceutical Co.,Ltd. | 2015060803 |
| Xiyanping_injection | 2ml:50mg | JiangxiQingfeng Pharmaceutical Co.,Ltd. | 2015071703 |
| Xiyanping_injection | 2ml:50mg | JiangxiQingfeng Pharmaceutical Co.,Ltd. | 2015071703 |
| Xiyanping_injection | 2ml:50mg | JiangxiQingfeng Pharmaceutical Co.,Ltd. | 2015080303 |
| Xiyanping_injection | 2ml:50mg | JiangxiQingfeng Pharmaceutical Co.,Ltd. | 2015072603 |
| Xiyanping_injection | 2ml:50mg | JiangxiQingfeng Pharmaceutical Co.,Ltd. | 2015080303 |
| Xiyanping_injection | 2ml:50mg | JiangxiQingfeng Pharmaceutical Co.,Ltd. | 2015082103 |
| Xiyanping_injection | 2ml:50mg | JiangxiQingfeng Pharmaceutical Co.,Ltd. | 2015083103 |
| Xiyanping_injection | 2ml:50mg | JiangxiQingfeng Pharmaceutical Co.,Ltd. | 2015090703 |
| Xiyanping_injection | 2ml:50mg | JiangxiQingfeng Pharmaceutical Co.,Ltd. | 2015090703 |
| Xiyanping_injection | 2ml:50mg | JiangxiQingfeng Pharmaceutical Co.,Ltd. | 2015090703 |
| Xiyanping_injection | 2ml:50mg | JiangxiQingfeng Pharmaceutical Co.,Ltd. | 2015091603 |
| Xiyanping_injection | 2ml:50mg | JiangxiQingfeng Pharmaceutical Co.,Ltd. | 2015091603 |
| Xiyanping_injection | 2ml:50mg | JiangxiQingfeng Pharmaceutical Co.,Ltd. | 2015091603 |
| Xiyanping_injection | 2ml:50mg | JiangxiQingfeng Pharmaceutical Co.,Ltd. | 2015102403 |
| Xiyanping_injection | 2ml:50mg | JiangxiQingfeng Pharmaceutical Co.,Ltd. | 2015102403 |
| Xiyanping_injection | 2ml:50mg | JiangxiQingfeng Pharmaceutical Co.,Ltd. | 2015111903 |
| Xiyanping_injection | 2ml:50mg | JiangxiQingfeng Pharmaceutical Co.,Ltd. | 2015111903 |
| Xiyanping_injection | 2ml:50mg | JiangxiQingfeng Pharmaceutical Co.,Ltd. | 2015111903 |
| Xiyanping_injection | 2ml:50mg | JiangxiQingfeng Pharmaceutical Co.,Ltd. | 2015111903 |
| Xiyanping_injection | 2ml:50mg | JiangxiQingfeng Pharmaceutical Co.,Ltd. | 2015111903 |
| Xiyanping_injection | 2ml:50mg | JiangxiQingfeng Pharmaceutical Co.,Ltd. | 2015122603 |
| Xiyanping_injection | 2ml:50mg | JiangxiQingfeng Pharmaceutical Co.,Ltd. | 2015122603 |
| Xiyanping_injection | 2ml:50mg | JiangxiQingfeng Pharmaceutical Co.,Ltd. | 2016011303 |
| Xiyanping_injection | 2ml:50mg | JiangxiQingfeng Pharmaceutical Co.,Ltd. | 2016011303 |
| Xiyanping_injection | 2ml:50mg | JiangxiQingfeng Pharmaceutical Co.,Ltd. | 2016011303 |
| Xiyanping_injection | 2ml:50mg | JiangxiQingfeng Pharmaceutical Co.,Ltd. | 2016022503 |
| Xiyanping_injection | 2ml:50mg | JiangxiQingfeng Pharmaceutical Co.,Ltd. | 2016022503 |
| Xiyanping_injection | 2ml:50mg | JiangxiQingfeng Pharmaceutical Co.,Ltd. | 2016022503 |
| Xiyanping_injection | 2ml:50mg | JiangxiQingfeng Pharmaceutical Co.,Ltd. | 2016041503 |
| Xiyanping_injection | 2ml:50mg | JiangxiQingfeng Pharmaceutical Co.,Ltd. | 2016041503 |
| Xiyanping_injection | 2ml:50mg | JiangxiQingfeng Pharmaceutical Co.,Ltd. | 2016042403 |
| Xiyanping_injection | 2ml:50mg | JiangxiQingfeng Pharmaceutical Co.,Ltd. | 2016042403 |
| Xiyanping_injection | 2ml:50mg | JiangxiQingfeng Pharmaceutical Co.,Ltd. | 2016042403 |
| Xiyanping_injection | 2ml:50mg | JiangxiQingfeng Pharmaceutical Co.,Ltd. | 2016052503 |
| Xiyanping_injection | 2ml:50mg | JiangxiQingfeng Pharmaceutical Co.,Ltd. | 2016052503 |
| Xiyanping_injection | 2ml:50mg | JiangxiQingfeng Pharmaceutical Co.,Ltd. | 2016060603 |
| Xiyanping_injection | 2ml:50mg | JiangxiQingfeng Pharmaceutical Co.,Ltd. | 2016060603 |
| Xiyanping_injection | 2ml:50mg | JiangxiQingfeng Pharmaceutical Co.,Ltd. | 2016060603 |
| Xiyanping_injection | 2ml:50mg | JiangxiQingfeng Pharmaceutical Co.,Ltd. | 2016063003 |
| Xiyanping_injection | 2ml:50mg | JiangxiQingfeng Pharmaceutical Co.,Ltd. | 2016063003 |
| Xiyanping_injection | 2ml:50mg | JiangxiQingfeng Pharmaceutical Co.,Ltd. | 2016063003 |
| Xiyanping_injection | 2ml:50mg | JiangxiQingfeng Pharmaceutical Co.,Ltd. | 2016063003 |
| Xiyanping_injection | 2ml:50mg | JiangxiQingfeng Pharmaceutical Co.,Ltd. | 2016063003 |
| Xiyanping_injection | 2ml:50mg | JiangxiQingfeng Pharmaceutical Co.,Ltd. | 2016081003 |
| Xiyanping_injection | 2ml:50mg | JiangxiQingfeng Pharmaceutical Co.,Ltd. | 2016081003 |
| Xiyanping_injection | 2ml:50mg | JiangxiQingfeng Pharmaceutical Co.,Ltd. | 2016091103 |
| Xiyanping_injection | 2ml:50mg | JiangxiQingfeng Pharmaceutical Co.,Ltd. | 2016091103 |
| Xiyanping_injection | 2ml:50mg | JiangxiQingfeng Pharmaceutical Co.,Ltd. | 2016091103 |
| Xiyanping_injection | 2ml:50mg | JiangxiQingfeng Pharmaceutical Co.,Ltd. | 2016101103 |
| Xiyanping_injection | 2ml:50mg | JiangxiQingfeng Pharmaceutical Co.,Ltd. | 2016101103 |
| Xiyanping_injection | 2ml:50mg | JiangxiQingfeng Pharmaceutical Co.,Ltd. | 2016101103 |
| Xiyanping_injection | 2ml:50mg | JiangxiQingfeng Pharmaceutical Co.,Ltd. | 2016120703 |
| Xiyanping_injection | 2ml:50mg | JiangxiQingfeng Pharmaceutical Co.,Ltd. | 2016120703 |
| Xiyanping_injection | 2ml:50mg | JiangxiQingfeng Pharmaceutical Co.,Ltd. | 2016122503 |
| Xiyanping_injection | 2ml:50mg | JiangxiQingfeng Pharmaceutical Co.,Ltd. | 2016122803 |
| Xiyanping_injection | 2ml:50mg | JiangxiQingfeng Pharmaceutical Co.,Ltd. | 2016122803 |
| Xiyanping_injection | 2ml:50mg | JiangxiQingfeng Pharmaceutical Co.,Ltd. | 2017022103 |
| Xiyanping_injection | 2ml:50mg | JiangxiQingfeng Pharmaceutical Co.,Ltd. | 2017022103 |
| Xiyanping_injection | 2ml:50mg | JiangxiQingfeng Pharmaceutical Co.,Ltd. | 2017022103 |
| Xiyanping_injection | 2ml:50mg | JiangxiQingfeng Pharmaceutical Co.,Ltd. | 2017030803 |
| Xiyanping_injection | 2ml:50mg | JiangxiQingfeng Pharmaceutical Co.,Ltd. | 2017032403 |
| Xiyanping_injection | 2ml:50mg | JiangxiQingfeng Pharmaceutical Co.,Ltd. | 2017030803 |
| Xiyanping_injection | 2ml:50mg | JiangxiQingfeng Pharmaceutical Co.,Ltd. | 2017032403 |
| Xiyanping_injection | 2ml:50mg | JiangxiQingfeng Pharmaceutical Co.,Ltd. | 2017030803 |
| Xiyanping_injection | 2ml:50mg | JiangxiQingfeng Pharmaceutical Co.,Ltd. | 2017032403 |
| Xiyanping_injection | 2ml:50mg | JiangxiQingfeng Pharmaceutical Co.,Ltd. | 2017032403 |
| Xiyanping_injection | 2ml:50mg | JiangxiQingfeng Pharmaceutical Co.,Ltd. | 2017032403 |
| Xiyanping_injection | 2ml:50mg | JiangxiQingfeng Pharmaceutical Co.,Ltd. | 2017040103 |
| Xiyanping_injection | 2ml:50mg | JiangxiQingfeng Pharmaceutical Co.,Ltd. | 2017040103 |
| Xiyanping_injection | 2ml:50mg | JiangxiQingfeng Pharmaceutical Co.,Ltd. | 2017040103 |
| Xiyanping_injection | 2ml:50mg | JiangxiQingfeng Pharmaceutical Co.,Ltd. | 2017040103 |
| Xiyanping_injection | 2ml:50mg | JiangxiQingfeng Pharmaceutical Co.,Ltd. | 2017040103 |
| Xiyanping_injection | 2ml:50mg | JiangxiQingfeng Pharmaceutical Co.,Ltd. | 2017040103 |
| Xiyanping_injection | 2ml:50mg | JiangxiQingfeng Pharmaceutical Co.,Ltd. | 2017061603 |
| Xiyanping_injection | 2ml:50mg | JiangxiQingfeng Pharmaceutical Co.,Ltd. | 2017061603 |
| Xiyanping_injection | 2ml:50mg | JiangxiQingfeng Pharmaceutical Co.,Ltd. | 2017061603 |
| Xiyanping_injection | 2ml:50mg | JiangxiQingfeng Pharmaceutical Co.,Ltd. | 2017061603 |
| Xiyanping_injection | 2ml:50mg | JiangxiQingfeng Pharmaceutical Co.,Ltd. | 2017061603 |
| Xiyanping_injection | 2ml:50mg | JiangxiQingfeng Pharmaceutical Co.,Ltd. | 2017061603 |
| Xiyanping_injection | 2ml:50mg | JiangxiQingfeng Pharmaceutical Co.,Ltd. | 2017061603 |
| Xiyanping_injection | 2ml:50mg | JiangxiQingfeng Pharmaceutical Co.,Ltd. | 2017040103 |
| Xiyanping_injection | 2ml:50mg | JiangxiQingfeng Pharmaceutical Co.,Ltd. | 2017040103 |
| Xiyanping_injection | 2ml:50mg | JiangxiQingfeng Pharmaceutical Co.,Ltd. | 2019041803 |
| Xiyanping_injection | 2ml:50mg | JiangxiQingfeng Pharmaceutical Co.,Ltd. | 2019041803 |
| Xiyanping_injection | 2ml:50mg | JiangxiQingfeng Pharmaceutical Co.,Ltd. | 2019041803 |
